# Perforated ulcer at the gastrojejunal anastomosis: a rare complication of pancreaticoduodenectomy (case report)

**DOI:** 10.11604/pamj.2024.48.38.43787

**Published:** 2024-05-31

**Authors:** Mohamed El Hammouti, Amine Majdoubi, Anass EL Achchi, Tariq Bouhout, Badr serji

**Affiliations:** 1 Surgical Oncology Department, Regional Oncology Center, Mohammed VI University Hospital, Oujda, Morocco,; 2 Mohammed First University Oujda, Faculty of Medicine and Pharmacy Oujda, Oujda, Morocco

**Keywords:** Gastrojejunal anastomosis ulceration, cephalic duodenopancreatectomy, spleen-preserving surgery, case report

## Abstract

Pancreaticoduodenectomy (PD) is recognized as one of the most intricate abdominal surgical procedures, often accompanied by high morbidity rates. The occurrence of an anastomotic ulcer at the gastrojejunal anastomosis post-pancreaticoduodenectomy surgery is a relatively uncommon complication, albeit potentially leading to severe, life-threatening consequences. The predominant symptomatology manifests as acute abdominal pain accompanied by peritonitis. Conventionally, diagnosis is achieved through computed tomography (CT) scans, facilitating subsequent management, and surgical management is recommended in the majority of instances. Herein, we present a rare case of a patient who experienced ulcer perforation at the gastrojejunal anastomosis site after undergoing pancreaticoduodenectomy with stomach preservation, and we reviewed the available literature to gain more comprehension of this rare complication of this type of surgical intervention.

## Introduction

Gastrojejunal anastomosis ulceration is not a widely recognized complication following cephalic duodenopancreatectomy (CPD). A recent study indicated that the incidence of marginal ulcers after CPD was 2.4%, with onset typically occurring at a median of 15.5 months post-surgery, although the occurrence of perforation has yet to be definitively established [1]. Our case exhibited several factors conducive to ulcer development at the anastomosis site, including the underlying disease, premature cessation of antisecretory medications, self-administration of non-steroidal anti-inflammatory drugs (NSAIDs) without medical oversight, decreased appetite, and fasting preceding the perforation event. Through an in-depth analysis of their clinical progression, this report aims to elucidate the pathophysiology underlying ulcerative perforation of the gastrojejunal anastomosis post-pancreaticoduodenectomy (DPC) and to provide insights for both preventive measures and therapeutic interventions.

## Patient and observation

**Patient information:** a 50-year-old patient with an unremarkable medical history was under our department's care for suspected lower choledochal thickening. In 2018, the patient underwent cephalic duodeno-pancreatectomy. Histopathological analysis of the surgical specimen showed no evidence of tumor cell proliferation, despite thorough sampling of the main bile duct and ampulla of Vater. The resection margins of the gastric and duodenal choledochal areas were unremarkable, and there was no tumor involvement observed in the retro portal lamina, with no lymph node invasion (7N-/7N). The postoperative period was uneventful. During hospitalization, the patient received analgesics, a 14-day course of antibiotics, and double-dose injectable proton pump inhibitors (PPIs). One week after surgery, the patient was discharged with one week of oral antibiotics, analgesics, and a single-dose oral PPI.

**Clinical findings:** in August 2023, the patient presented with acute abdominal pain. On clinical examination, the patient was hemodynamically and respiratorily stable, with tenderness in the epigastric region. No weight loss or change in bowel habits.

**Figure 1:**
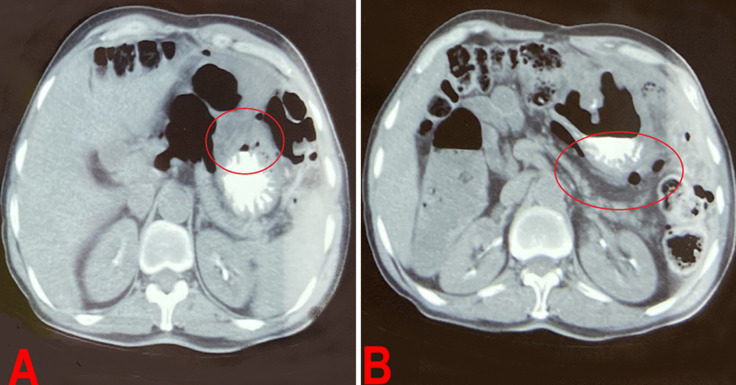
A, B) abdominal and pelvic computed tomography scan showing generalized peritonitis due to perforation of a hollow organ (circles)

**Diagnostic assessment:** an abdominopelvic CT scan revealed generalized peritonitis due to the perforation of a hollow organ. There was no contrast extravasation after opacification, but there was suspicion of a gastric defect opposite the area of the gastrojejunal anastomosis, given the predominant infiltration and presence of extra-digestive air bubbles at this level. Additionally, there was suspicious thickening of the parietal area at the site of the gastrojejunal anastomosis, along with mesenteric lymphadenopathy (Figure 1).

**Figure 2:**
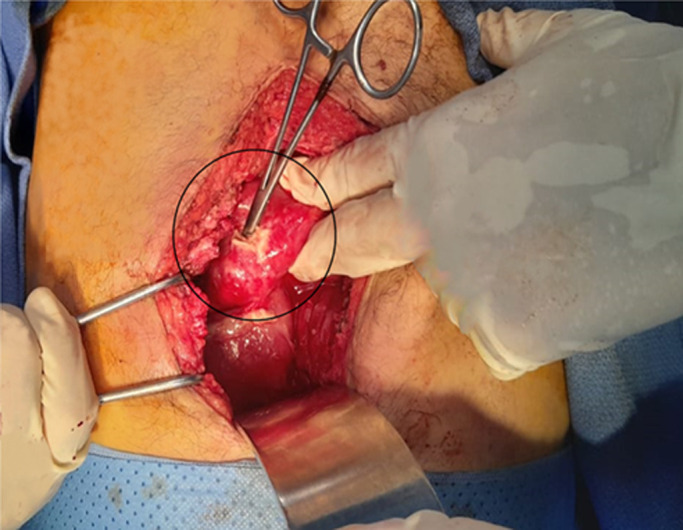
per-operative image of perforated ulcer (circle) at the gastrojejunal anastomosis

**Therapeutic intervention:** following the diagnosis, the patient underwent emergency exploratory laparotomy with saline lavage, suturing of the stomach breach, and drainage (Figure 2).

**Follow-up and outcomes:** postoperative care proceeded without complications. The patient was discharged after five days with a prescription for analgesics, a one-week course of antibiotics, and double-dose proton pump inhibitors (PPIs) for one month. The thoracoabdomino pelvic scan and physical examination demonstrated that there were no recurrences after a follow-up of six months.

**Patient perspective:** the patient declined to give his perspective on her illness.

**Informed consent:** written informed consent was obtained from the patient for publication and any accompanying images.

## Discussion

The etiology of marginal peptic ulceration following DPC is thought to be linked to the altered gastrointestinal anatomy post-surgery, alongside traditional factors including gastric acidity, Non-Steroidal Anti-Inflammatory Drug use (NSAID use), prolonged stress, and Helicobacter pylori infection [2]. Various acid-inhibiting hormones, such as secretin, gastric inhibitory polypeptide, and vasoactive intestinal peptide, are predominantly synthesized in the small intestine, particularly near the stomach [3]. In animal studies comparing duodenal resection with non-resection [2], the resection group exhibited 5.7 and 3.6 times higher gastric acid secretion levels under fasting and fed conditions, respectively. Hence, the removal of the duodenum and upper jejunum may expedite gastric acid production. Additionally, the duodenum is rich in Brunner's glands, which secrete an alkaline-rich mucinous fluid. However, the jejunal limb used for reconstruction following DPC lacks these glands. Consequently, the jejunal mucosa may not adequately neutralize gastric acid, thereby creating a more ulcerogenic environment [3].

In the reported case, ulceration at the gastrojejunal anastomosis occurred after surgery preserving part of the stomach, which retained its inherent gastric acid secretion, heightening the risk of ulcer development in the anastomosed jejunum [4]. Several pathophysiological mechanisms can be proposed to explain the specific ulcerogenic potential associated with this surgical procedure: i) the elongated intestinal loop separating the pancreas and bile ducts from the gastric outlet may lead to excessive absorption of alkaline fluid from the intestine, reducing the amount available for neutralizing gastric acid at the gastrojejunostomy; ii) inadequate gastric resection; iii) postoperative pancreatic duct stenosis, often subtle and insidious, may nullify the neutralizing capacity of secretions; iv) gastric inhibitory polypeptide, a recently isolated intestinal polypeptide with incompletely understood actions, can inhibit gastric acid secretion in response to histamine, pentagastrin, and insulin hypoglycemia [4]; v) resection of the antrum, a significant portion of the pancreas, and the entire duodenum can disrupt hormonal balance, potentially triggering an ulcerogenic response. The time interval between primary surgery and ulcer perforation onset varies among patients. Studies suggest that fasting, as observed in our case, may accelerate marginal ulcer perforation development, although further investigation is warranted to validate this hypothesis [5].

Moreover, the incidence of marginal ulcer perforation varies from that of the primary disease. Unlike pancreatic cancer patients, those with biliary cancer often retain pancreatic secretory function, potentially leading to more effective gastric acid neutralization. The underlying reasons for these paradoxical findings remain unclear and warrant further investigation through the accumulation of case series [6]. In our case, the patient underwent surgical treatment with direct closure of the lesion, although conservative management may be appropriate for select patients. Additionally, chronic administration of antisecretory agents is considered beneficial in preventing ulcer recurrence [7]. The clinical course of patients suggests that the management approach for marginal ulcer perforation may align with that for conventional peptic ulcer perforation [8]. However, it's important to note that the patient in this report has not undergone proper evaluation for Helicobacter pylori infection. The clinical course of the patients suggests that the management strategy for marginal ulcer perforation may be consistent with that for conventional peptic ulcer perforation. The patient presented in this report hasn't been properly evaluated for H. pylori infection [8].

## Conclusion

We present a patient who presented with ulcer perforation at the site of the gastrojejunal anastomosis after cephalic duodeno pancreatectomy. Exposure to excess acid due to lack of use of antisecretory agents and fasting was considered the primary cause of anastomotic site peptic ulcer perforation. Long-term chronic administration of antisecretory agents and avoidance of fasting are necessary in patients undergoing stomach-sparing DPC, particularly in cases of biliary disease or a history of peptic ulcer disease, to prevent marginal ulcer perforation.
